# A New Thermal Conductivity Model and Two-Phase Mixed Convection of CuO–Water Nanofluids in a Novel I-Shaped Porous Cavity Heated by Oriented Triangular Hot Block

**DOI:** 10.3390/nano10112219

**Published:** 2020-11-07

**Authors:** Amin Asadi, Maysam Molana, Ramin Ghasemiasl, Taher Armaghani, Mihail-Ioan Pop, Mohsen Saffari Pour

**Affiliations:** 1 Institute of Research and Development, Duy Tan University, Da Nang 550000, Vietnam; aminasadi@duytan.edu.vn; 2 Faculty of Natural Sciences, Duy Tan University, Da Nang 550000, Vietnam; 3 Department of Mechanical Engineering, Wayne State University, Detroit, MI 48202, USA; molana@wayne.edu; 4 Department of Mechanical Engineering, West Tehran Branch, Islamic Azad University, Tehran 14687-63785, Iran; ghasemiasl.r@wtiau.ac.ir; 5 Department of Mechanical Engineering, Mahdishahr Branch, Islamic Azad University, Mahdishahr 35618-75915, Iran; 6 Department of Mathematics, Babeş-Bolyai University, 400084 Cluj-Napoca, Brasov, Romania; popm.ioan@yahoo.co.uk; 7 Department of Mechanical Engineering, Shahid Bahonar University of Kerman, Kerman 76169-13439, Iran; mohsensp@kth.se

**Keywords:** nanofluids, mixed convection, cooling, entropy generation, thermal conductivity, correlation

## Abstract

This paper investigates the cooling performance of nanofluid (NF) mixed convection in a porous I-shaped electronic chip with an internal triangular hot block using Buongiorno’s two-phase model. This type of cavity and hot block geometry has not been studied formerly. The NF was assumed to be a mixture of water and CuO nanoparticles (NP) up to 4% of volume concentration. As most published mathematical models for the thermal conductivity of NF give inaccurate predictions, a new predictive correlation for effective thermal conductivity was also developed with a high accuracy compared to the experimental data. The results showed that any increase in the NP volume concentration enhances the average Nusselt number ($\bar{Nu}$) and the normalized entropy generation, and reduces the thermal performance of the cavity in all orientations of the hot block. The maximum enhancement in cooling performance was 17.75% and occurred in the right-oriented hot block in the sand-based porous cavity. Furthermore, adding the NP to the base fluid leads to a more capable cooling system and enhances the irreversibility of the process.

## 1. Introduction

Conventional cooling fluids typically yield relatively low performance and therefore they are unable to remove the desirable heat that the new industrial processes and equipment are generating. Today, most industries need a more efficient method of heat removal than the past. Thermal scientists have devised several innovative methods for cooling applications during the twentieth century, including but not limited to heat sinks, heat pipes, different types of heat exchangers, and so on. Nevertheless, none of them could attract the attention of the scientific community as much as the nanofluids (NFs). NFs are relatively new type of heat transfer fluids with promising thermophysical properties. The ever-increasing number of the scientific publications and conferences as well as the industrial projects on the NF demonstrates the importance of this new category of cooling fluids.

NFs were introduced for the first time by Choi and Eastman [1] to enhance heat transfer of a cooling process. NFs are suspension of nanoscale particles in conventional heat transfer fluids like water, oil and ethylene glycol. This will enhance the thermophysical properties like density, viscosity, and thermal conductivity. Generally, NPs are synthesized from metals or oxides, as well as carbon nanotubes.

NF applications have been tried in a wide range of industrial processes and equipment including but not restricted to heat exchangers [2,3,4,5,6], nuclear reactors [7], medicine [8], automobiles [9,10], electronic chip cooling [11], renewable energies [12,13], heating and tempering processes [14,15,16,17], lubrication [18,19], combustion [20], etc. However, there are some other promising cooling technologies, which have attracted the attention of research communities, including but not restricted to phase change materials (PCMs) [21], electrocaloric effects [22], etc.

Enclosures also have different applications in a wide range of industries and equipment, such as cooling [23,24], Heating, Ventilation, and Air Conditioning (HVAC) [25], nuclear power [26], heat exchangers [27,28], renewable energies [29], etc.

Patterson and Imberger [30] simulated a differentially heated cavity under an unsteady natural convection. They also used a scale analysis to show that a number of initial flow types are possible. They realized that even though the steady state doesn’t influence by the value of the Prandtl number, it is evident that the transient flows may depend strongly on this number.

Tiwari and Das [31] studied the heat transfer in a two-sided lid-driven square cavity using the NF, implementing a finite volume approach using the Semi-Implicit Method for Pressure Linked Equations (SIMPLE) algorithm. It has been found that the variation of the average Nusselt number $\bar{(Nu})$ is nonlinear with the solid volume fraction and the NPs are able to change the flow pattern of a fluid from free convection to the forced convection regime.

Chamkha et al. [32] studied the effects of heat sink and source and entropy generation on the Magnetohydrodynamics (MHD) mixed convection of copper-water NF in a lid-driven square porous enclosure with partial slip, numerically. Their results showed that the enhancement of the Hartmann number decreases the average Nu decreases considerably and NPs increase the entropy generation.

Al-Rashed et al. [33] et al. studied the effects of a hot elliptical centric cylinder, cavity angle and NP volume concentration in an inclined lid-driven cavity filled with water–alumina NF subjected to a mixed convection, numerically. They concluded that the Nu reduces with any increase in the Richardson number (Ri).

Mehmood et al. [34] investigated the alumina–water NF filled square porous cavity using a Koo-Kleinstreuer-Li (KKL) model in a mixed convection to examine how a nonlinear thermal radiation and inclined magnetic field can affect the heat and fluid field. To this end, a Darcy–Brinkman–Forchheimer extended model was employed to formulate the governing differential equations. They observed a growth in the maximum stream function with a rise in porosity and Da for a fixed value of Ri. They also found out an augmentation in maximum stream function value with a growth in thermal radiation parameter for free convection flows.

Baroon et al. [35] used a two-phase approach to simulate mixed convection and entropy generation in a lid-driven cavity with rotating cylinders filled by NF under a magnetic field. They found out that the heat transfer enhances with any reduction in the Hartmann number and Ri and any increase in the NP volume concentration. The presence of the cylinder and its angular velocity also improved the heat transfer. In addition, isothermal cylinders had a great impact on increasing heat transfer.

MHD mixed convection of the NF in an open C-shaped cavity was studied by Armaghani et al. [36]. Their enclosure was under constant magnetic field. NF entered the enclosure from the top right corner and exited from it from the bottom right corner. Eventually, they observed that with any increase in the aspect ratio of the enclosure, the heat transfer increases the local Nu by increasing the Ri.

The influence of partial slip on entropy generation and the MHD mixed convection in a square lid-driven porous enclosure saturated with a Cu–water NF was investigated by Chamkha et al. [37], numerically. Their results showed an augmentation in the heat generation/absorption parameter decreases the Nu. Also, when the volume fraction is raised, the Nu and entropy generation are reduced.

Armaghani et al. [38] studied numerically the water–alumina NF natural convection heat transfer and entropy generation in a baffled L-shaped cavity. They showed that increasing the Hartmann number reduces the entropy generation; however, the thermal performance increases. Increasing the aspect ratio raises heat transfer and thermal performance.

Mixed convection phenomena in a rectangular NF filled, non-Darcian porous enclosure with various wall speed ratios was studied by Nithyadevi et al. [39]. They implemented The Finite Volume Method (FVM) to solve transport equations for fluid and heat fields. In addition, the porous matrix was supposed to be rigid, containing with spherical, isotropic and homogeneous particles. They observed that dispersion of CuO NPs can enhance the heat transfer rate dramatically due to the impact of large thermal conductivity of the solid–fluid mixture in NF.

To the best knowledge of the authors, the heat transfer phenomenon of the NF in the I-shaped electronic chip has not been studied comprehensively. This paper investigates mixed convection heat transfer in an I-shaped porous enclosure filled with NF including an internal high temperature triangular solid block numerically using finite volume method. Two different porous media, sand and compact metallic powder, as well as different orientations of the internal hot block have been investigated. The CuO NP volume concentration in water was set in the range of 0 to 4 percent. This study implements Buongiorno’s method with some assumptions, including laminar, incompressible, steady-state, homogenous, Newtonian NF, and a thermal equilibrium between NPs and the base fluid’s molecules. To ensure that the prediction of effective thermal conductivity is accurate, a new predictive mathematical model with an acceptable accuracy is proposed. Using the second law of thermodynamics, entropy analysis, and thermal performance criteria, the best situation of the cavity consideration was determined.

## 2. Mathematic Modeling and Governing Equations

### 2.1. Problem Geometry

**Figure 1:**
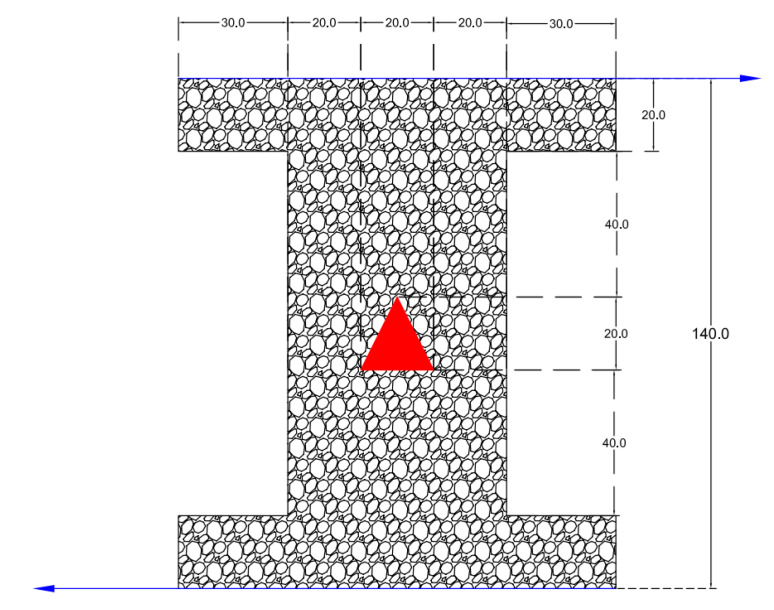
The problem geometry including a porous media with an internal hot block. All dimensions are in millimeters.

The geometry of the problem is shown in Figure 1. This was a lid-driven (bottom and upper lids) porous I-shaped enclosure saturated with water–CuO NF. The bottom and upper walls were moving in the right and left directions with a constant velocity of 10 m/s, respectively. All other walls were insulated. The temperatures of the cold and hot walls were set to 306 and 346 degrees Kelvin, respectively. As shown, the enclosure was made from porous media and included a hot triangular block at the geometric center. All shown scales are in centimeters. The problem was solved with four different cases of the hot block’s orientation. The porous media also included two different materials: sand and compact metallic powder, which were considered separately.

**Figure 2:**
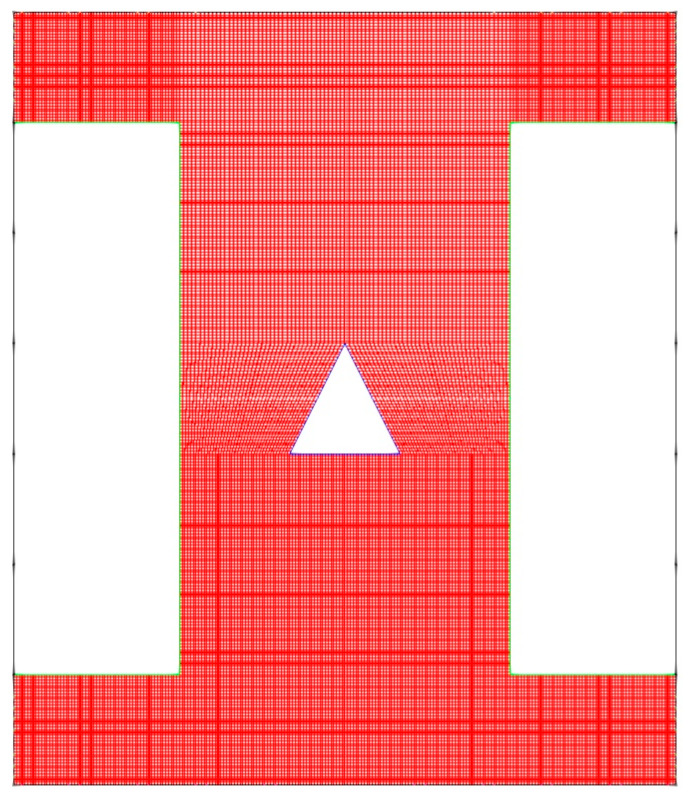
Mesh network for triangular hot block.

Figure 2 demonstrates the mesh network for the problem. Generally, all cases were meshed with two cells per millimeter. However, the number of cells per millimeter changed dramatically near to the hot block. It should be noted that the total cells of the mesh for up, down, left and right were 36,800, 48,000, 40,000 and 40,000, respectively.

### 2.2. Material

The considered NF was CuO (copper oxide) produced by Iranshimi Company, Urmia, Iran. Such NPs are suspended in water as the base fluids up to 4% range of volume concentration. The mean diameter and NPs was assumed to be 29 nanometers and no surfactant was considered. Table 1 presents the reference values for different thermophysical properties of CuO NPs and water at the ambient conditions. The porous media were also considered to be made from sand and metallic powder with thermal conductivity of 3 W/m.K and 60 W/m.K, respectively. These two cases were solved separately. The porosity and diffusivity of the porous media for two cases were set to 0.4 and 10^−8^ m^2^, respectively.

### 2.3. Governing Equations

Before going forward to describe the solution method, we need to take a look at the assumptions as follows: steady fluid flow, incompressible flow, laminar flow, two-dimensional flow, no chemical reactions, no viscous dissipation, no radiative heat transfer, and homogenous porous media.

To investigate the quality of the slip between the NPs and the base fluid’s molecules, the conservation equations of mass and momentum are as follows:

The conservation of mass:

(1) $$\nabla .\vec{V}=0$$

The conservation of momentum:

(2) $$\nabla \left(\rho_{nf}\vec{V}\times \vec{V}\right)=-\left(\nabla \rho_{nf}\right)g.h-\nabla \left(P\right)+S$$

where *S* is the source term for porous media and is defined as:

(3) $$S=-\left(\mu_{nf}\times d\times \vec{V}+\frac{\rho_{nf}\times f\times \vec{V}}{2}\right)$$

where *d* and *f* are the Darcy coefficient and Forchheimer coefficient, respectively. They are defined as follows [42]:

(4) $$d=\frac{\varepsilon }{K}$$

(5) $$f=\frac{3.5}{\sqrt{150}\times \sqrt{K}\times \varepsilon^{0.5}}$$

The conservation of energy for NF in a porous medium is as:

(6) $$\rho_{nf}\times (1-\beta_{nf}\left(T-T_{0}\right)\times C_{p,nf}\times V.\nabla T=\nabla \left(k_{eff}\right).\nabla T+\varepsilon \rho_{nf}C_{p,np}\times \left[D_{B}\nabla \varphi \nabla T+D_{T}\frac{\nabla T.\nabla T}{T}\right]$$

The NP volume concentration of NF should satisfy the equation below [42], in which $\vec{V}$, *ε*, *k_eff_*, To and *β* are the vector of Darcy velocity, porosity, effective thermal conductivity of the porous media, the average temperature of the NF, and thermal expansion coefficient, respectively. Also, *C_p,nf_*, *φ*, *D_B_*, *D_T_*, ρ, *C_p,np_* and *μ* are specific heat of NPs at the constant pressure, the NP volume concentration, Brownian diffusion coefficient, thermophoresis coefficient, density of the NF, specific heat of NF at the constant pressure and dynamic viscosity of NF, respectively.

(7) $$\frac{1}{\varepsilon }\vec{V}.\nabla \varphi =\nabla .\left[D_{B}\nabla \varphi +\frac{\nabla T}{T}\nabla \varphi \right]$$

where the Brownian diffusion coefficient and thermophoresis coefficient are defined by Equations (8) and (9), respectively [43,44]. In these equations, *K_b_*, *T*, *d_np_*, *k_bf_* and *k_np_* represent the Boltzmann constant, NF bulk temperature, mean diameter of the NPs, thermal conductivity of the base fluid and thermal conductivity of NPs, respectively.

(8) $$D_{B}=\frac{K_{b}T}{3\pi \pi_{nf}d_{np}}$$

(9) $$D_{T}=\left(\frac{0.26k_{bf}}{2k_{bf}+k_{np}}\right)\left(\frac{\mu_{nf}}{\rho_{nf}}\right)\varphi$$

We can calculate the total entropy generation using the Equation (10) [45].

(10) $$S_{gen}=\left(\frac{k_{eff}}{T_{0}^{2}}\right)\times \left({\left(\frac{\partial T}{\partial x}\right)}^{2}+{\left(\frac{\partial T}{\partial y}\right)}^{2}\right)+\frac{\mu_{nf}}{KT_{0}}\times \left(u^{2}+v^{2}\right)+\frac{\mu_{nf}}{T_{0}}\times \left(2\left({\left(\frac{\partial u}{\partial x}\right)}^{2}+{\left(\frac{\partial u}{\partial y}\right)}^{2}\right)\right)+\left(\frac{\partial u}{\partial x}+\frac{\partial v}{\partial y}\right)$$

Integrating the effective thermal conductivity to the thermal conductivity of the base fluid times temperature gradient gives the average *Nu* as follows [45]:

(11) $$\bar{Nu}=\int -\left(\frac{k_{eff}}{k_{bf}}\right)\times \left(\frac{\partial \theta }{\partial n}\right)dn$$

where, *n* is the direction perpendicular to the surface. Dimensionless temperature also is defined as Equation (12).

(12) $$\theta =\frac{T-T_{cold}}{T_{hot}-T_{cold}}$$

To calculate the effective thermal conductivity of the porous media, we used the proportional equation as follows:

(13) $$k_{eff}=\varepsilon k_{nf}+\left(1-\varepsilon \right)k_{solid}$$

where, *k_solid_* and *k_nf_* are the thermal conductivity of porous material and thermal conductivity of the NF. The density of NF also can be calculated as follows:

(14) $$\rho_{nf}=\varphi \rho_{np}+\left(1-\varphi \right)\rho_{bp}$$

where, $\rho_{np}$ and $\rho_{bf}$ are the density of NPs and the density of the base fluid, respectively. To calculate the effective dynamic viscosity of NF, we used an empirical correlation [46]:

(15) $$\frac{\mu_{nf}}{\mu_{bf}}=1.475-0.319\varphi +0.051\varphi^{2}+0.009\varphi^{3}$$

The thermal expansion coefficient of NF could be determined using Equation (16).

(16) $$\rho_{nf}\beta_{nf}=\left(1-\varphi \right)\rho_{bf}+\varphi \rho_{np}\beta_{np}$$

To find the enhancement achieved by the NF we used some concepts, including normalized *Nu*, normalized entropy generation, and thermal performance of the cavity [47], which are shown in Equations (17)–(19).

(17) $$Nu^{*}=\frac{Nu}{Nu_{0}}$$

(18) $$S^{*}=\frac{S_{gen}}{S_{gen,\;0}}$$

(19) $$\eta =\frac{S_{gen}}{Nu}$$

where, Nu*, S*, *η*, $Nu_{0}$, $S_{gen,\;0}$ are the normalized *Nu*, normalized generation entropy, thermal performance of the cavity, the Nu of the base fluid, and entropy generation of the base fluid, respectively.

## 3. Proposing a New Correlation for CuO–Water Thermal Conductivity

To calculate the thermal effectivity of NF, we proposed a new predictive correlation based on the experimental data in the literature. To do so, we extracted 35,441 data on copper oxide-based NF thermal conductivity from the experimental studies [48,49,50,51] in order to correlate a new model. Then, we used the Gauss–Newton multivariable regression method and, after 31 iterations, the proposed Equation (20) resulted:

(20) $$k_{eff}=k_{bf}\times \left(1+8.86068\times \varphi \times {\left(\frac{0.738066}{d_{p}}\right)}^{2}\right)$$

where, *k_eff_*, *k_bf_*, *φ*, and *d_p_* are effective thermal conductivity of NF (W/m.K), thermal conductivity of base fluid (W/m.K), NP volume concentration (%), and NP mean diameter (nm), respectively.

**Figure 3:**
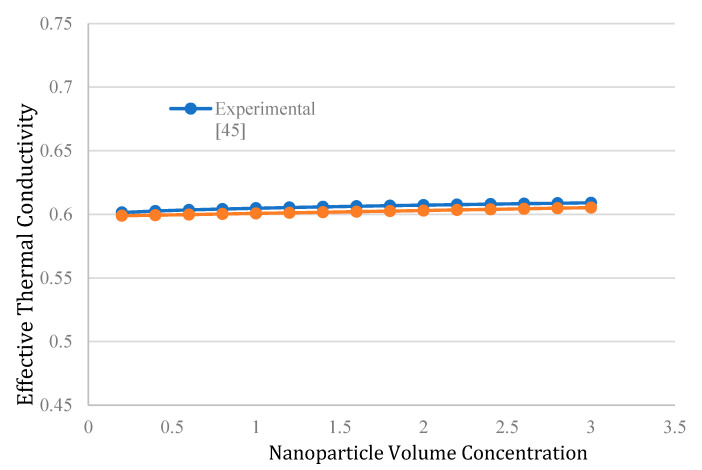
The comparison of the experimental data and predicted effective thermal conductivity by the proposed correlation against the NP volume concentration.

The average absolute error of the proposed correlation compared to the experimental data is 2.71%. To make sure about the validations of the proposed correlation, the predicted values are compared with an experimental study (Patel et al. [48]) represented in Figure 3. As shown, both data (experimental and predicted) show increasing values for the effective thermal conductivity with an increase in the NP volume concentration.

## 4. Numerical Method and Validation

### Code Validation

**Figure 4:**
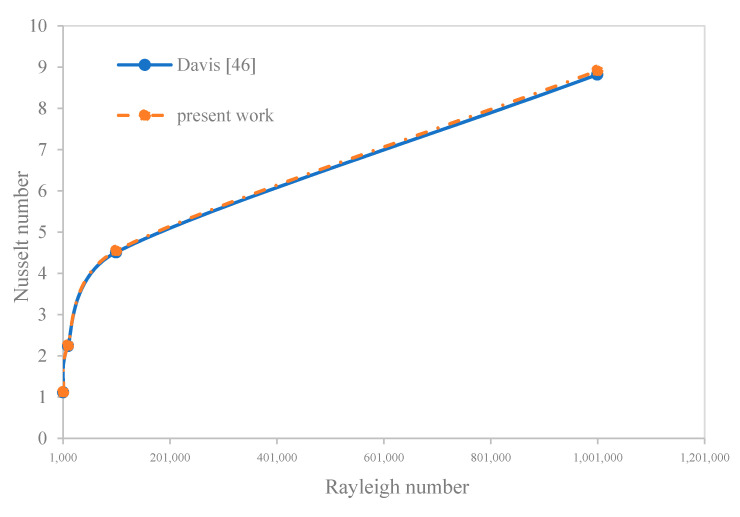
The comparison of the results of the present work with reference.

The numerical method of the present work was validated in two steps with well-known references. First of all, we compared the results for air flowing in the cavity with the results of Davis [40]. Figure 4 shows the variations of the Nu versus the Ra (Ra) number in order to compare the results of the numerical solution with the reference. The results demonstrate that the maximum error is 1.066%. Therefore, the numerical solution of the present work is as accurate at the first step.

Then, we compared the results of the NF natural convection in a porous cavity with the experimental work of Nithiarasu et al. [42]. Table 2 shows the results of our numerical solution and comparison with the reference. It is clear that the present work gives very accurate results and the maximum error from the reference value is 1.069%. The error also decreases with any increase in the Ra number and porosity.

An extensive mesh testing procedure was conducted using seven different meshes to obtain the appropriate grid size for the solution of the present problem considering constant parameters including the NP volume concentration and Ra number. Table 3 represents the obtained results for different size of meshes. Comparing the obtained numerical results of the average Nu, it was found that the value of the average Nu for 67,600 (260 × 260) elements showed a slight difference (0.32%) from the results calculated for other elements. Therefore, the grid size of 6218 nodes and 67,600 elements met the requirements of accurate solution with a proper grid independency for the present work.

## 5. Results and Discussion

**Figure 5:**
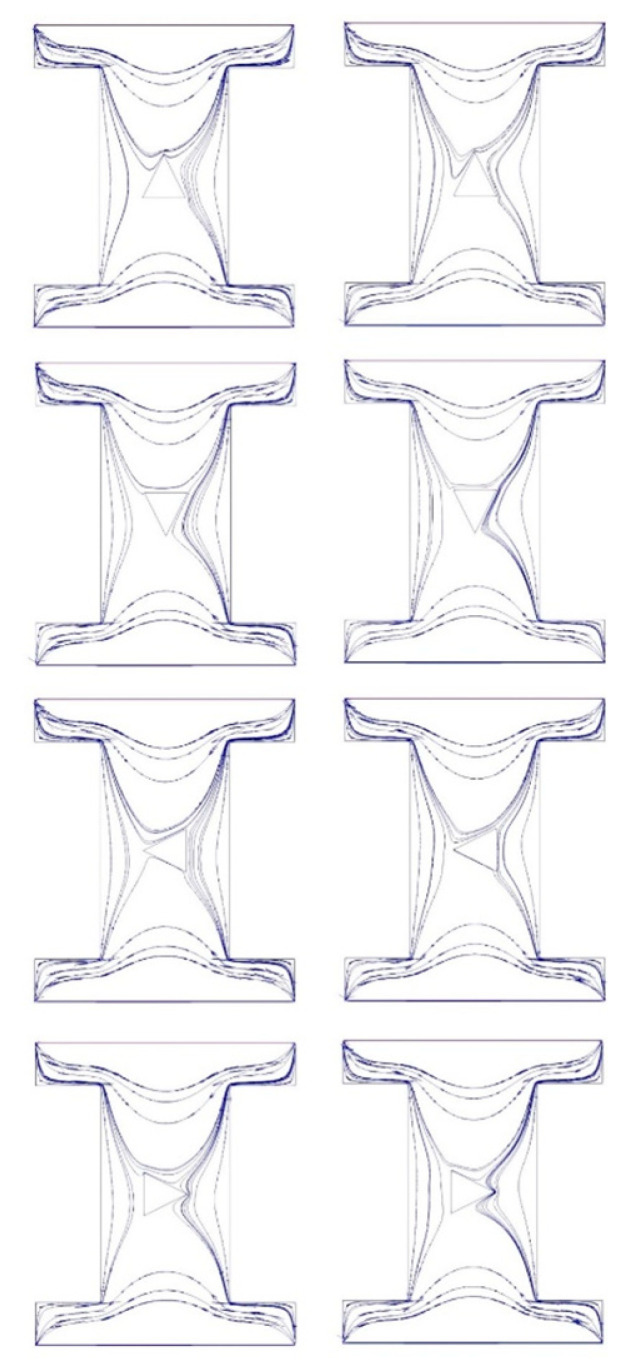
The streamline contours of the 4% vol. nanofluid (NF) in sand-based (**left column**) and metallic powder-based cavities (**right column**) with different orientations of the hot block.

The obtained results will be presented in this section, including four orientations of the triangle and two porous media for different NP volume concentration from zero to 4%. Figure 5 shows the streamline contours of the 4% vol. NF in sand-based (left column) and metallic powder-based cavities (right column) with different orientations of the hot block. It seems that the contours are affected by the orientation of the hot block, especially for the left orientation. There are also more compact streamline contours for the NF in the metallic powder-based cavity.

**Figure 6:**
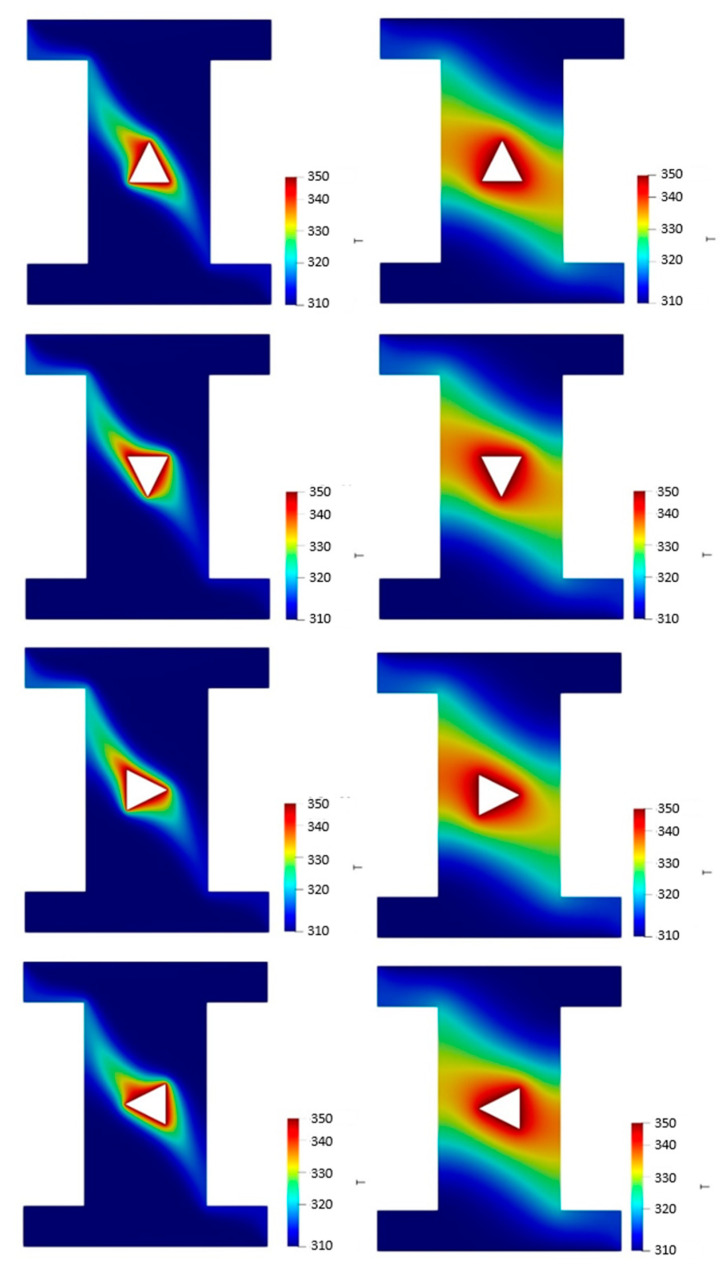
The temperature contours of the 4% vol. NF in sand-based (**left column**) and metallic powder-based cavities (**right column**) with different orientations of the hot block.

Figure 6 also represents the temperature contours of the 4% vol. NF in sand-based (left column) and metallic powder-based (right column) cavities with different orientations of the hot block. It is found that the rotation of the hot block affects the temperature contours in both porous cavities. Generally, there are higher temperatures in the metallic powder-based cavity than the sand-based cavity, due to the large difference in their thermal conductivities.

**Figure 7:**
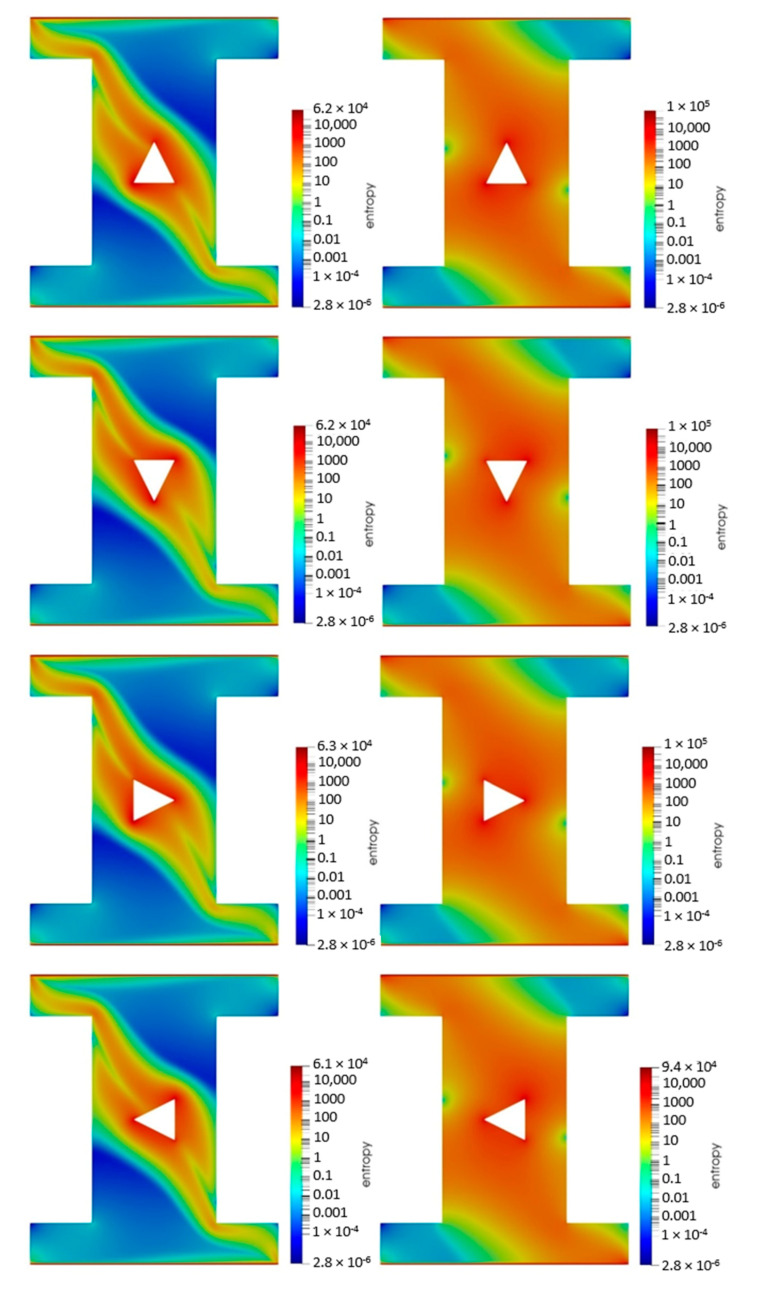
Entropy generation of the 4% vol. NF in two different cavities including the sand-based (**left column**) and the metallic powder-based (**right column**) porous cavities with different orientations of the hot block.

The entropy generation contours of the 4% vol. water-CuO NF in two different cavities including the sand-based (left column) and the metallic powder-based (right column) porous cavities with different orientations of the hot block are shown in Figure 7. It is clear that the entropy generation contours were affected by the different orientations of the hot block. However, changing the orientation did not change considerably the amount of the entropy and the elongation of the entropy contours. It should be noted that the entropy generation contours for the metallic powder-based porous cavity were denser but more uniform than those of the sand-based porous cavity, in accordance with the temperature contours.

**Figure 8:**
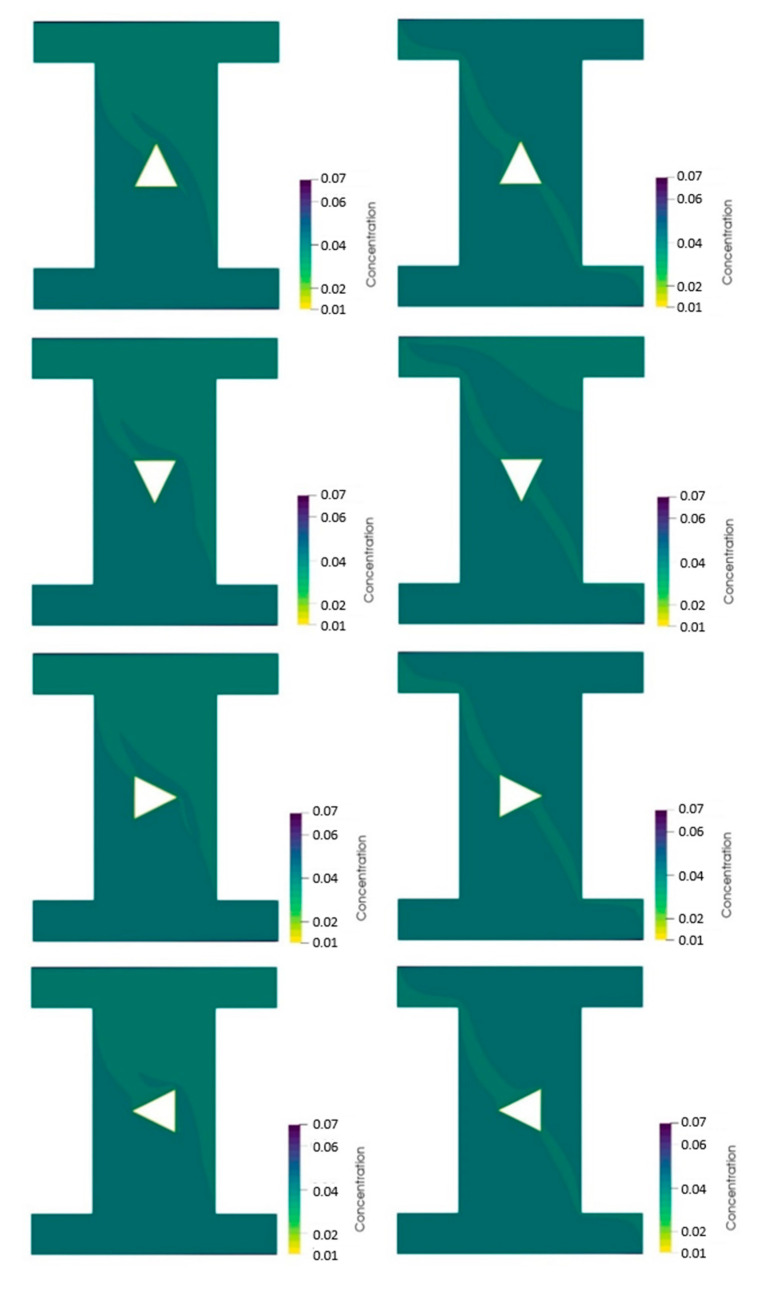
NP distribution contours of the 4% vol. NF in two different cavities including the sand-based (**left column**) and the metallic powder-based (**right column**) porous cavities with different orientations of the hot block.

Figure 8 shows the NP distribution contours of the 4% vol. NF in two different cavities including the sand-based (left column) and the metallic powder-based (right column) porous cavities with different orientations of the hot block. The NP distribution contours in the sand-based porous cavity show that there were more NPs below the hot block. The orientation of the hot block didn’t change considerably the NP distribution contours, except for the left orientation. The different orientations also didn’t change the NP distribution contours for the metallic powder-based porous cavity, except for the down orientation, which led to lower amount of NPs at the upper section of the cavity.

**Figure 9:**
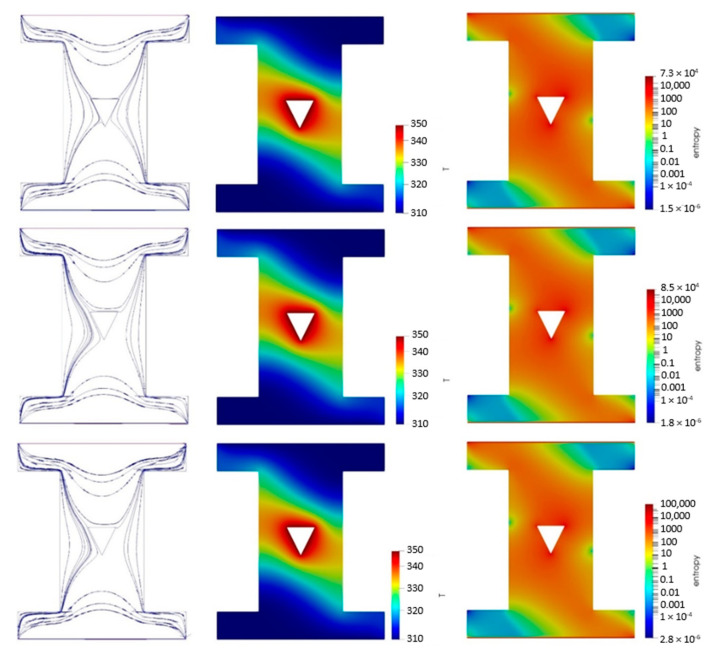
The streamlines (**left**), temperature (**middle**) and entropy generation (**right**) contours of the NF in the metallic powder-based porous cavity for the down-oriented hot block and the NP volume concentrations of 0% (**the first row**), 2% (**the middle row**) and 4% (**the bottom row**).

Figure 9 shows the streamlines, temperature and entropy generation contours of the NF in the metallic powder-based porous cavity in the left, middle, and right columns, respectively for the down-oriented hot block. The first, second and last row of Figure 9 is considered for the base fluid, 2% vol. and 4% vol. NF, respectively. It is shown that the streamlines were distributed symmetrically around the hot block and within the cavity for the base fluid. The streamlines were also aggregated at the left side of the hot block for more NP volume concentrations. Interestingly, there was no considerable difference between the temperature and the entropy generation contours for different NP volume concentrations.

**Figure 10:**
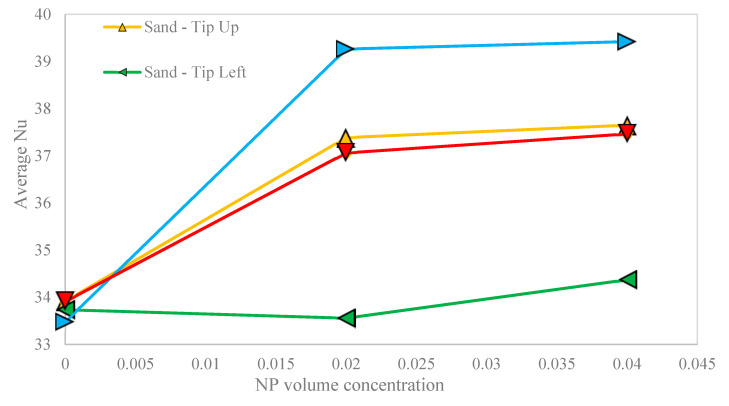
The variation of the average Nu versus the NP volume concentration for the sand-based porous cavity and different orientations of the hot block.

The average Nu as a function of the NP volume concentration and the orientation of the hot block for the sand-based porous cavity is shown in Figure 10. Generally, the average Nu rose continually with any increase in the NP volume concentration. However, the average Nu and its enhancement were higher for the right-oriented (17.75%), followed by up-oriented (11.00%), down-oriented (10.48%) and left-oriented (1.88%) hot block, respectively. Nevertheless, in all orientations of hot block, there was no considerable enhancement in the average Nu from 2% to 4% of the NP volume concentration, except the left-oriented hot block.

**Figure 11:**
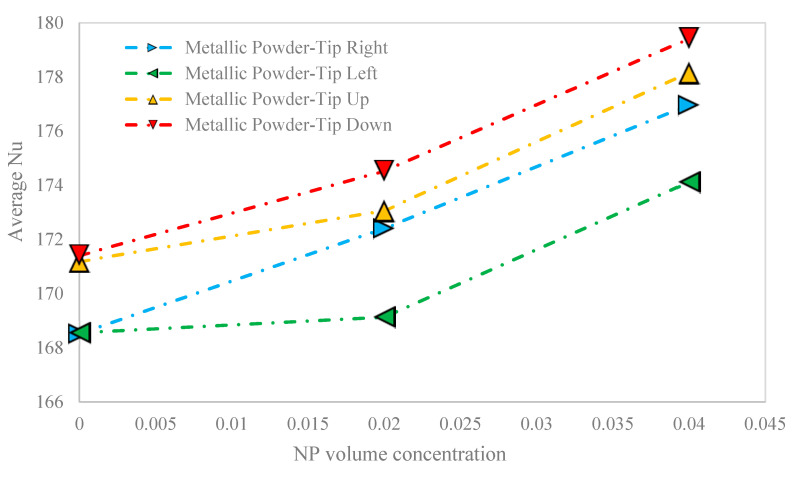
The variation of the average Nu versus the NP volume concentration for the metallic powder-based porous cavity and different orientations of the hot block.

The variation of the average Nu versus the NP volume concentration for the metallic powder-based porous cavity and different orientations of the hot block is shown in Figure 11. Any increase in the NP volume concentration enhanced the average Nu, in all orientations of the hot block. Nevertheless, the enhancement in the average Nu was the maximum for the right-oriented hot block with 5.01%, followed by the down-oriented (4.85%), up-oriented (4.07%), and left-oriented (3.30%) hot block. It should be noted that the amount of the average Nu for the metallic powder-based porous cavity was roughly four times more than those of the sand-based porous cavity. The enhancement in the average Nu for the sand-based porous cavity was much more than those of the metallic powder-based porous cavity.

**Figure 12:**
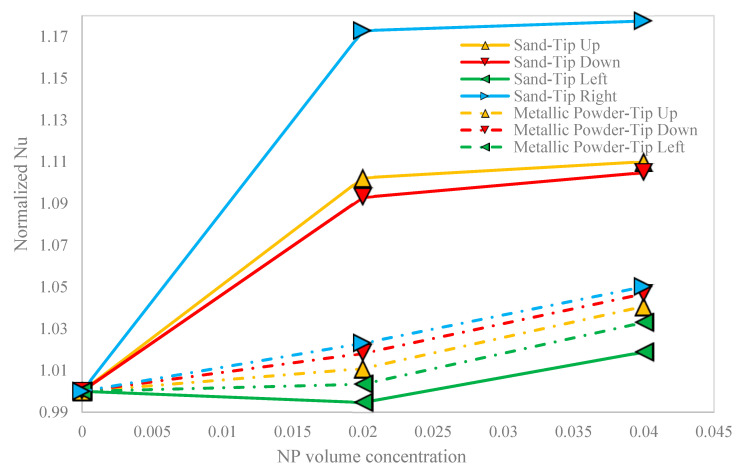
The variation of the normalized Nu of the NF versus the NP volume concentration, in all cases.

The variation of the normalized Nu of the NF versus the NP volume concentration, in all cases is shown in Figure 12. The normalized Nu is defined as the ratio of the Nu to the Nu of the base fluids at the same conditions. It is obvious that there was a direct relationship between the normalized Nu and the NP volume concentration, in all cases. The right-oriented hot block in the sand-based porous cavity yielded the higher value of the normalized Nu in all NP volume concentrations. Meanwhile, the left-oriented hot block gave the lowest normalized Nu in the two different porous cavities.

**Figure 13:**
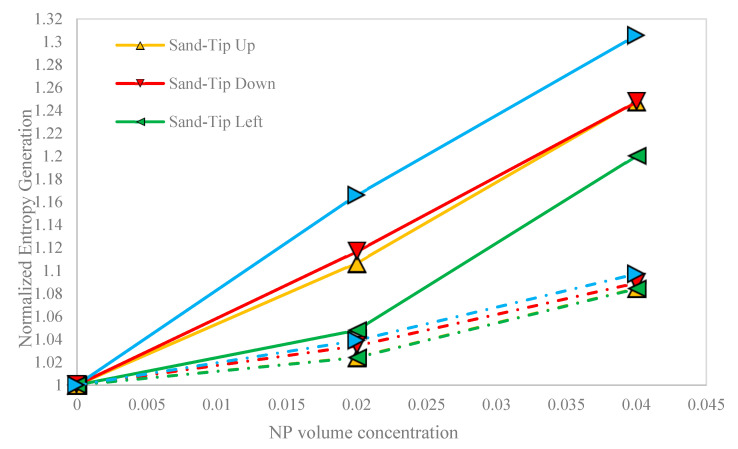
Normalized entropy generation against the NP volume concentration, in all cases.

Figure 13 shows the normalized entropy generation against the NP volume concentration, in all cases. The normalized entropy generation is defined as the ratio of the entropy generation of the considered case to the entropy generation of the base fluid at the same conditions. Generally, any increase in the NP volume concentration led to a rise in the normalized entropy generation in all cases. Typically, the sand-based porous cavity gave more normalized entropy generation and, therefore, more irreversibility in the process. The right-oriented hot block in the sand-based porous cavity also yielded the highest value of the normalized entropy generation (1.035).

**Figure 14:**
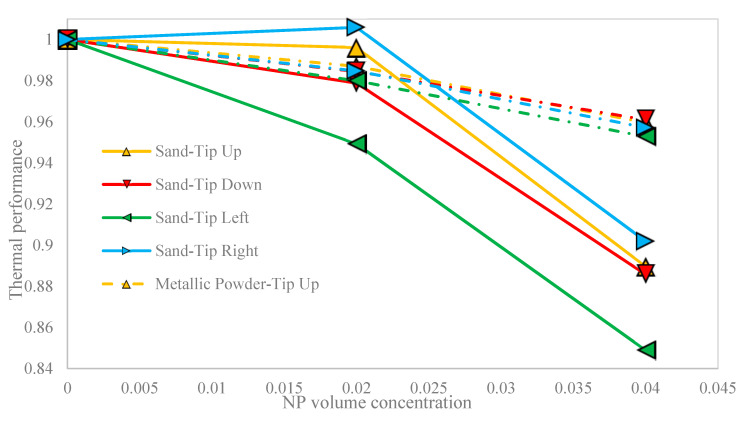
The thermal performance of the different porous cavities against the NP volume concentration in all cases.

Figure 14 also shows the thermal performance of the different porous cavities against the NP volume concentration in all cases. Thermal performance is the ratio of the normalized Nu versus the NP volume concentration and a higher thermal performance means that the Nu is achieved with less irreversibility. As it is shown, thermal performance of two porous cavities decreased with any increase in the NP volume concentration. It means that the cavity experienced more irreversibility in the process for NF than the base fluid to achieve the same Nu. Although adding the NP to the base fluid led to more Nu, it enhanced the irreversibility of the process. However, the maximum reduction in the thermal performance occurred in the left-oriented hot block in the sand-based porous cavity.

## 6. Conclusions

The NF mixed convection in a porous I-shaped cavity was investigated numerically. The NF was assumed to be a mixture of water and CuO NPs up to 4% of volume concentration. A finite volume method was employed to solve the governing equations of eight cases as the combinations of four orientations of the internal triangular hot block, and two porous media: sand and compact metallic powder.

A new predictive correlation for effective thermal conductivity of the NF is proposed using the multivariable regression method based on the experimental data. It is shown that the proposed correlation predicted the effective thermal conductivity of the NF with a very low error compared to the experimental data. The major findings of the study are as follows:

1. The rotation of the hot block affected the temperature and entropy generation contours in both porous cavities.
2. Any increase in the NP volume concentration enhanced the average Nu, the cooling performance and the normalized entropy generation, and reduced the thermal performance of the cavity in all orientations of the hot block.
3. The average Nu for the metallic powder-based porous cavity was roughly four times more than that of the sand-based porous cavity.
4. The enhancement in the average Nu for the sand-based porous cavity was much more than that of the metallic powder-based porous cavity. The maximum enhancement in the average Nu was 17.75%.
5. The right-oriented hot block in the sand-based porous cavity yielded the higher value of the normalized Nu in all NP volume concentrations, while the left-oriented hot block gave the lowest normalized Nu in the two different porous cavities.
6. The sand-based porous cavity gave more normalized entropy generation and, therefore, more irreversibility in the process.
7. Adding the NP to the base fluid led to more Nu, enhancing the irreversibility of the process. However, the maximum reduction in the cooling performance occurred in the left-oriented hot block in the sand-based porous cavity.
8. The best and worst cooling performance was achieved with the 2% vol. NF in the sand-based right-oriented internal hot block and 4% vol. NF in the sand-based left-oriented internal hot block.

## Figures and Tables

**Table 1:** Thermophysical properties of copper oxide nanoparticles (NPs) and water at the ambient conditions [40,41].

Material	Thermal Expansion (1/K)	Viscosity (N.S/m^2^)	Thermal Conductivity (W/m.K)	Density (kg/m^3^)	Heat Capacity (J/kg.K)
CuO	8.5 × 10^−6^	-	18	6500	540
Water	2.07 × 10^−4^	1.002 × 10^−3^	0.598	998.3	4179

**Table 2:** The comparison of the results of the present study with the reference.

Test Number	Ra Number	Da	Porosity	Reference Nu [40]	Nu	Error
1	10^3^	10^−2^	0.4	1.008	1.0208	1.069%
2	10^6^	10^−4^	0.6	2.725	2.74106	0.589%
3	10^4^	10^−2^	0.9	1.64	1.65509	0.92%

**Table 3:** Grid independency test of the present work.

Test Number	NP Volume Concentration (%)	Ra Number	Reference Nu [44]	Mesh Size (Element Number)	Clock Time (Seconds)	Obtained Nu	Variation (%)
1	3	5.6 × 10^7^	29.0769	140 × 140	334	29.2141	-
2	3	5.6 × 10^7^	29.0769	160 × 160	651	28.8535	1.23
3	3	5.6 × 10^7^	29.0769	180 × 180	940	28.5944	0.89
4	3	5.6 × 10^7^	29.0769	200 × 200	1457	28.4005	0.67
5	3	5.6 × 10^7^	29.0769	220 × 220	2209	28.2551	0.51
6	3	5.6 × 10^7^	29.0769	240 × 240	3781	28.1401	0.40
7	3	5.6 × 10^7^	29.0769	260 × 260	4247	28.0485	0.32
